# Hg Pollution Indices along the Reis Magos River Basin—Brazil: A Precursory Study

**DOI:** 10.3390/ijerph191912626

**Published:** 2022-10-03

**Authors:** Eldis Maria Sartori, Bruna Miurim Dalfior, Carolina Scocco Provete, Suellen Geronimo Cordeiro, Maria Tereza Weitzel Dias Carneiro, Maria de Fátima Fontes Lelis, Gilberto Fonseca Barroso, Geisamanda Pedrini Brandão

**Affiliations:** 1 Laboratory of Atomic Spectrometry (LEA)—Chemistry Department, Federal University of Espírito Santo, Vitória 29075-910, Brazil; 2 Water Laboratory—Chemistry Department, Federal University of Espírito Santo, Vitória 29075-910, Brazil; 3 Limnology and Environmental Planning Laboratory (LimnoLab)—Department of Oceanography and Ecology, Federal University of Espírito Santo, Vitória 29075-910, Brazil

**Keywords:** mercury monitoring, environmental samples, direct determination, pollution indices

## Abstract

Mercury is a metal present in the Earth’s crust, but due to human contribution, its concentration can increase, causing environmental impacts to aquatic ecosystems, among others. The Reis Magos River Hydrographic Basin represents economic and socio-environmental importance for the state of Espírito Santo, Brazil. However, there are not many publications regarding the quality of water and sediments, so no data is reported concerning the total concentration of Hg. Thus, the present work aimed to evaluate the distribution of total Hg in water and sediments along this hydrographic basin. For a better inference, physicochemical parameters of the water were determined (temperature, pH, electrical conductivity, oxidation-reduction potential (ORP), turbidity, dissolved oxygen (DO), total dissolved solids (TDS), and salinity), and in the sediments, the contents of matter organic matter, pH, carbonates and granulometry. Mercury determination was performed by Thermodecomposition and Amalgamation Atomic Absorption Spectrometry (TDA AAS) with a DMA-80 spectrometer. The Hg determined in the water was lower than the limit of quantification, 0.14 µg∙L^−1^, which is lower than the maximum limits recommended by world reference environmental agencies. In the sediment samples, the Hg found were below 170 µg∙kg^−1^, values below which there is less possibility of an adverse effect on the biota. However, when the degree of anthropic contribution was evaluated using the Geoaccumulation index (IGeo), the contamination factor (CF), and the ecological risk potential index (E_F_), there was evidence of moderate pollution. Thus, this highlighted the need for monitoring the region since climatic variations and physical-chemical parameters influence the redistribution of Hg between the water/sediment interface.

## 1. Introduction

Demographic, industrial and agricultural expansions that occur unplanned have had harmful consequences for the environment in the vicinity of rivers and seas. Deforestation, silting, mining, inadequate disposal of solid waste, and effluent releases can cause the redistribution of chemical elements into the ecosystem, thus compromising the biota, altering the elemental composition of sediments, reducing the quality of water in rivers, as well as causing damage to human health [1]. Among the potentially toxic chemical elements, mercury is considered one of the ten most harmful elements to public health [2].

The toxicity of As, Cd, Pb, and Hg against host microorganisms in the human intestine, responsible for maintaining the immune system, food fermentation, and vitamin production, was investigated [3]. Among the potentially toxic elements, mercury showed greater toxicity than other elements when observed in bacteria naturally found in the human intestine. According to the authors, there is limited information on the binding of Hg to gut bacteria.

The harmfulness of mercury comes from its great capacity for bioaccumulation and biomagnification. The first occurs when the body absorbs mercury faster than it can be excreted. The second occurs when there is an increase in the metal concentration at each level of the food chain. This expressive capacity occurs because mercury forms complexes with the body’s proteins, reducing its metabolic functions [4]. This ease of reaching the food chain occurs since mercury can be found in the environment mainly in inorganic form, as elemental mercury and in chloride and sulfur salts, or in organic form as methyl or ethyl mercury [5].

The seasonal trends towards mercury bioaccumulation and toxic effects in Asian clam (*Corbicula fluminea*) and microbial communities from sediment collected in the estuary of the Hyeongsan River, South Korea, were investigated. The authors observed that the concentration of total mercury and methylmercury in the bottom sediments was higher in winter and lower in summer. Conversely, water concentrations were higher in summer than in winter, and mercury biomagnification in clams was higher in the summer [6].

In aquatic environments, mercury, of anthropic origin, is generally incorporated into the sediment in its inorganic form, which, due to variations in the physical-chemical characteristics of the environment, can undergo methylation, giving rise to methylmercury [7]. Therefore, the concern with mercury in environments such as rivers and seas becomes relevant in the scientific community [8].

In evaluating the aquatic ecosystem, using the sediment matrix to determine total mercury reveals the contamination process. The increase in the total mercury concentration in the sediments linked to pH and water temperature variations can directly influence the bioavailability of this element in the water and, consequently, in coastal vegetation and microbiota, thus reaching the food chain. Therefore, the routine monitoring and control of this element in aquatic environments are crucial [9].

Although the human contribution of Hg to the environment is mainly caused by the use of Hg in gold extraction and the burning of fossil fuels, contamination of the hydrographic basin can also result from atmospheric deposition, inadequate waste disposal, as well as leaching of agricultural soil [10,11,12,13]. Thus, although studies are generally focused on regions with activities with more significant impacts, it is essential to investigate regions without clear point sources of Hg contamination, looking for areas potentially contaminated by diffuse sources of Hg or with specific characteristics for mobilization and bioaccumulation of this element [14,15]. Studies of this nature favor the understanding of the geochemical behavior of Hg, as well as the monitoring, spatial distribution, and perception of possible enrichment pathways [16].

Particular attention should be given to environments that, in addition to presenting diffuse economic activities in their surroundings, are used for water distribution, such as the case of the Reis Magos River (RMR). Located in Southeastern Brazil, in the state of Espírito Santo, the Reis Magos River Basin (RMRB) covers an area of 671 km^2^ with land use and land cover dominated by agriculture (i.e., coffee, rice, and beans), animal husbandry, and seven urban areas [17]. The region’s soil presents oxisol characteristics and is chemically and mineralogically related to the local lateritic crust, mainly derived from gibbsite and oxyhydroxides, and made of kaolinite, gibbsite, quartz, and goethite, a crystalline structure prone to the retention of trace elements, such as mercury.

This river water has been used for irrigation, livestock, and human consumption [18]. The RMRB represents economic and socio-environmental importance for the state of Espírito Santo. However, water and sediment quality reports are nonexistent, particularly regarding mercury contamination. Since Hg is a potential contaminant with a severe impact on the aquatic food chain, and very few studies have been conducted in the RMRB, this study focused on determining total mercury content in the sediment and river water. This study aims for a better understanding of the distribution of Hg in the aquatic ecosystems of the fluvial system and its estuary, supporting the assessment of the fluvial environmental quality.

## 2. Materials and Methods

### 2.1. Study Area

Due to its ecological importance, the Reis Magos River Basin (RMRB) was used as a study environment. This area is considered a nursery for countless species of aquatic organisms and a feeding region for other animals; it also has economic importance, evidenced by the fishermen’s colony in the region [19]. In addition, the Reis Magos River has been used for water distribution for human supply since 2017 [18]. Despite its importance, there are not many publications about its quality [20,21,22], and there are no reference values (background) for mercury present in this body of water.

**Figure 1:**
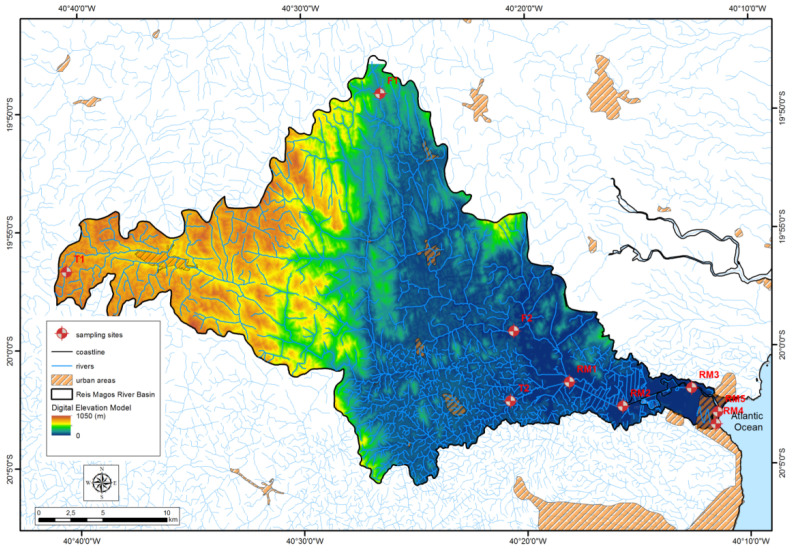
Reis Magos River Basin and their respective sampling stations.

The RMRB belongs to the Brazilian Hydrographic Basin of the Southeast Atlantic, in the north-central coastal region of the State of Espírito Santo, and is formed by the Fundão sub-basins to the north, Timbuí to the south, and Reis Magos as the main basin. It covers an area of approximately 671.8 km^2^, its primary source is located in Santa Teresa and flows into the municipality of Serra, and its extension covers the municipalities of Fundão, Ibiraçu, Santa Leopoldina, Santa Teresa, and Serra [17]. The region of the headwaters of the Reis Magos basin is marked by the presence of two formers, the Timbuí River, to the south, and the Fundão River, to the north, which runs parallel to each other, descending the slopes of the valleys as distinct sub-basins (Figure 1).

The use and land cover in the RMRB comprises 31.46% native forest, 25.74% pasture area, 9.46% reforestation (eucalyptus), 5.71% built-up area, 5.13% coffee, and 22.5% others, such as temporary cultivation, mangroves, sandbanks, sugarcane cultivation and mining [23].

Water and sediment samples were collected along the RMR and its tributaries, the Fundão River (F) and the Timbuí River (T), in August 2019 (S1: sampling 1—winter; dry) and January 2020 (S2: sampling 2—summer; rainy), and the sampling stations are indicated on the map in Figure 1. No sediments were collected at sampling stations T1 and F1 (river springs).

Table 1 shows the sampling stations, their respective characteristics, and geographic coordinates; negative values indicate west longitudes and south latitudes.

### 2.2. Collection, Preparation, and Analysis of Physical-Chemical Parameters of the Samples

Surface water samples were collected in duplicate for the determinations of total and dissolved Hg and conditioned in bottles previously decontaminated plastics, while the bottom sediment samples were collected on the surface manually and packed in plastic bags previously decontaminated. The samples were kept under refrigeration, at 4 °C, until analysis.

The sediment samples were dried to constant weight, at 60 °C, in an oven for drying (Ethik Technology, SP, BR). Then, they were homogenized, quartered, and sieved to a fraction smaller than 63 µm. Water samples were acidified to 7% *v*·*v*^−1^ with HCl for conservation and Hg analysis.

Mercury determination in water and sediment samples was performed by Thermodecomposition and Amalgamation Atomic Absorption Spectrometry (TDA AAS) with a DMA-80 spectrometer (Direct Mercury Analyzer, MILESTONE SRL, Italy). The operational parameters were described in the Supplemental Material.

The physical-chemical parameters of the water (temperature, pH, electrical conductivity, oxidation-reduction potential (ORP), turbidity, dissolved oxygen (DO), total dissolved solids (TDS), and salinity) were determined in situ using the Horiba U-50 portable multiparameter analyzer.

The measuring of sediment pH, carbonate, and organic matter contents followed the protocols in the literature [24,25,26]. The granulometric study of the sediments in their fractions (gravel + pebble, sand, silt, and clay) was carried out according to guidelines from the Embrapa (Brazilian Agricultural Research Corporation) Soil Analysis Methods Manual [27].

### 2.3. Contamination and Pollution Indices

To assess Hg contamination in the studied area, sediment and water quality guidelines and standards (SQG) stipulated by the Brazilian National Council for the Environment (CONAMA), Canadian Council of Ministers of the Environment (CCME), and USA’s National Oceanic and Atmospheric Administration (NOAA). Limit values are described in Table 2 [28,29,30].

There are guide values established by the CCME and NOAA for Hg in sediments from water, where TEL (Threshold Effect Concentration) indicates a concentration below which there is less possibility of an adverse effect on the biota, and PEL (Probable Effect Level) indicates the concentration level above which there is a greater probability of adverse effects to the biota [29,31].

Based on Canadian, North American, and European publications, the Brazilian National Council for the Environment implemented Resolution No. 454/2012, which regulates sediment quality and classifies the elements’ concentration in levels 1 and 2. Those levels represent, respectively, the threshold below which there are no adverse effects on the biota and the level above which there are effects on the biota (Table 2) [32].

Sediment contamination was also evaluated by determining the pollution indices: geoaccumulation, IGeo $\left(IGeo=\frac{C_{n}\;}{1.5\times B_{n}}\right)$ and the contamination factor, CF (CF=CnBn) [33,34,35,36]; where $C_{n}$ represents the concentration of Hg in the sample and $B_{n}$ the reference value of Hg content in the Earth’s crust. In this study, the reference value described by Wedepohl, equal to 56 µg∙kg^−1^ was used since there is no background value for the region [37]. In addition, the potential ecological risk index E_F_, a toxic response factor that takes into account the degree of element toxicity $\left(E_{F}=CF\cdot T\right)$ was calculated, where T represents the toxic response factor, equal to 40 for Hg [38].

The pollution indices evaluated (IGeo, CF, and EF) and their descriptions are shown in Table 3.

## 3. Results and Discussion

### 3.1. Results

#### 3.1.1. River Water Samples Analysis

The concentrations of total Hg determined in the water samples collected in the two samplings were below the LoQ (0.14 µg∙L^−1^). In addition, observing the values obtained for DO, pH, temperature, and turbidity in the water samples (Table S1).

#### 3.1.2. River Sediment Samples Analysis

The river sediment samples were analyzed, and the concentration values of Hg, pH, OM, and carbonate, as well as the granulometric fractions, are presented in Table S2 (Supplementary Material).

### 3.2. Discussion

#### 3.2.1. River Water Analysis

The water samples were analyzed, and the parameter results are in Table S1—Supplementary Material. Overall, there was a decrease in TDS in the rainy seasons (sampling 2). This substantial dilution was observed, as well as a drop in electrical conductivity [39]. During this sampling period, water turbidity increased due to the increase in river flow. River turbidity is dynamic, and its steady state is significantly altered during heavy rainfall events, which causes flooding, increases soil erosion, and drastically reduces water quality. Thus, turbidity becomes a significant concern as most health risks are associated with suspended organic matter, bacteria, and other microorganisms [40,41,42].

The concentrations of total Hg determined in the water samples collected in the two samplings were below the LoQ (0.14 µg∙L^−1^). However, as this value was lower than the values allowed by the water quality standards (Table 2), it is possible to infer that Hg concentrations in those water samples have a low potential to cause adverse effects on biota and human health.

In addition, observing the values obtained for DO, pH, temperature, and turbidity in the water samples (Table S1) and comparing them with the water quality indices (WQI) of the RMRB in the same period of collection, it is verified that they are consistent with the classification established by the “National Sanitation Foundation” (NSF) having a WQI from reasonable to good, indicating water suitable for human consumption after conventional treatment [43,44,45].

#### 3.2.2. River Sediment Analysis

**Figure 2:**
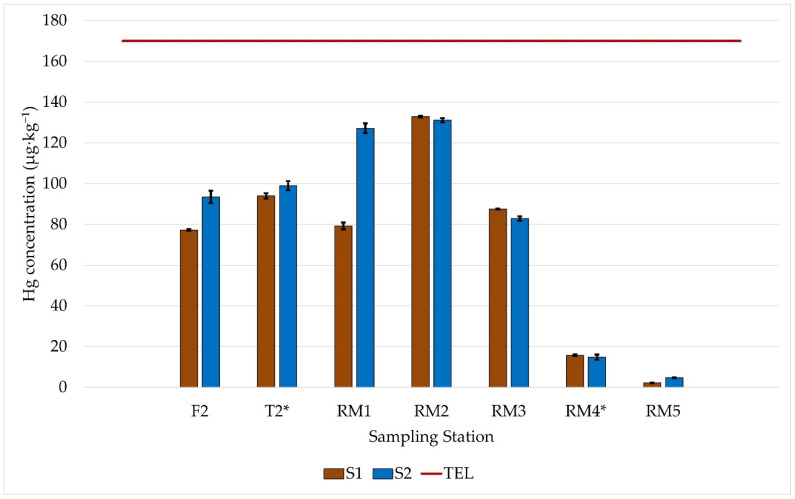
Hg concentration determined in sediment samples collected at different stations of the Rio Reis Magos River Hydrographic Basin in sampling S1 and S2. (-) Limit of concentration below which there is less possibility of an adverse effect on the biota (TEL). * No statistical differences with 95% confidence level of Hg concentration between the sampling periods.

Figure 2 shows the distribution of Hg concentration at the sampling stations on S1 (winter; dry) and S2 (summer; rainy).

The Student’s *t*-test was applied, with 95% confidence, between the averages obtained in the different sampling for the same sampling station. It was verified that there is no statistically significant difference between the averages, only in T2 and RM4. The most significant variation was found in the concentration of Hg in the sediment samples collected in RM1, where there is the confluence of the tributary rivers. In general, along the RMRB, there was an increase in the concentration of Hg in S2 to S1, probably due to the increasing rainfall, as shown in Figure S1 [46,47]. This may have enabled the sedimentary input from its tributaries and soil leaching from the surroundings. In addition, there may have been a disturbance in the river, causing sediment particles with higher Hg contents to be mobilized closer to the surface [6]. It is noted that RM2 presented the highest concentration of Hg, which may be associated with the highest levels of OM and fines content, which allow higher adsorption of metals since the higher surface contact of fine grains, and the significant contents of reactive geochemical substrates [48]. It was also verified that Hg contents decrease downstream towards the estuary, RM4, and RM5.

**Figure 3:**
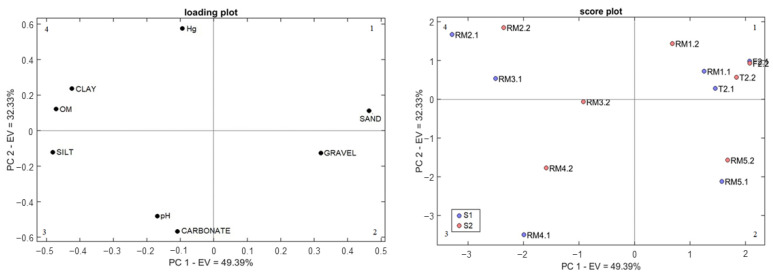
Principal component analysis with the physical-chemical characteristics of the sediment and Hg concentration.

The distribution of sediment samples according to the similarity of Hg, pH, OM, carbonate, and granulometric fractions is shown in the PCA model with a total of 81.72% of variance explained by the first two principal components (Figure 3). The samples were separated by principal component 1 (PC1), with 49.39% of the total variance of the data influenced by gravel, sand, silt, clay, and OM contents. Principal component 2 (PC2) explained 32.33% of the total variance, and the samples were separated according to Hg concentration, pH, and carbonate content.

In all samplings, there is a grouping of samples F2, T2, and RM1 in quadrant 1, probably because the Timbuí river (T2) and Fundão river (F2) converge in RM1. In quadrant 4, the progression of the samples in the score chart demonstrates a grouping of RM2 and RM3 due to a direct correlation with the OM, clay, and Hg concentration. It is essential to highlight that RM3 is a sampling station downstream of the ETE. These behaviors can be verified with the data obtained and recorded in Table S2, in which the Hg concentration tends to be directly proportional to the OM and clay content, as already verified in other published studies [15,49]. It is also observed that the carbonate content is correlated with the pH, being more evident in RM4 (quadrant 3).

**Figure 4:**
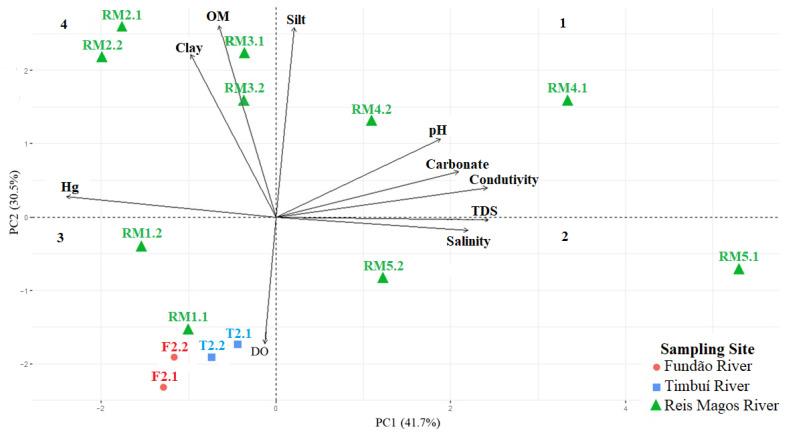
Principal component analysis with physical-chemical characteristics of sediment and water and Hg contents in sediment samples.

Correlating the determined concentrations of Hg in the sediment samples with the physical-chemical characteristics of the sampling stations, including the water samples, the formation of three groups among different temporal samplings was obtained by the Principal Component Analysis (PCA) (Figure 4).

The samples were separated by principal component 1 (PC1), with 41.7% of the total variance of the data influenced by the TDS content, conductivity, Hg concentration, salinity, carbonate content, and pH. Principal component 2 (PC2) explained 30.5% of the total variance, and the samples were separated according to OM content, silt content, clay content, and DO.

It can be seen in Figure 4 that the samples were separated into three groups. The first group is located in quadrants 1 and 2, with samples from stations RM4 and RM5, located at the lower RMR and next to urban areas. These samples with higher salinity (RM4 0.2 ppt in S1 and 0.2 ppt at S2; and RM5 0.3 ppt at S1 and 0.2 ppt at S2) showed the lowest Hg contents (RM4 15.73 ± 0.36 µg∙kg^−1^ in S1 and 14.9 ± 1.3 µg∙kg^−1^ in S2; and RM5 2.23 ± 0.11 µg∙kg^−1^ in S1 and 4.71 ± 0.18 µg∙kg^−1^ in S2). The pattern of lower Hg and other trace element contents in estuaries has been observed in other studies [50,51,52].

The second group is in quadrant 3, comprising samples from stations T2, F2, and RM1, with RM1 at the junction of the Timbuí and Fundão Rivers. In this way, we can infer similarity due to proximity. T2, F2, and RM1 were separated from the third group located in quadrant 4, mainly due to their high DO and low OM content, considering that in terms of Hg concentration, these groups have similar values. Sampling stations T2, F2, and RM1 receive the drainage of cropland areas, and the Hg content may be influenced by applying pesticides containing this element [53].

In the third group, located in quadrant 4, are the samples from stations RM2 and RM3. RM3 is located in an urban area downstream of a wastewater treatment plant (WTP), with high OM and low DO contents. Agricultural activities also influence the RM2 station, and as it is located in a rural area, it may be receiving the release of effluents into the water, which may be influencing the OM and DO contents. At this sampling station, the highest concentrations of Hg were found in sediment samples from both samplings, 132.89 ± 0.47 µg∙kg^−1^ in S1 and 131.1 ± 1.1 µg∙kg^−1^ in S2. However, the Hg contents were below the maximum allowable limits established by the CONAMA and CCME (Table 1 and Figure 2).

Regarding Hg concentrations in all sediment samples (Figure 2), there were no levels above the TEL limit, below which there is less possibility of an adverse effect on the aquatic biota [29,32].

**Figure 5:**
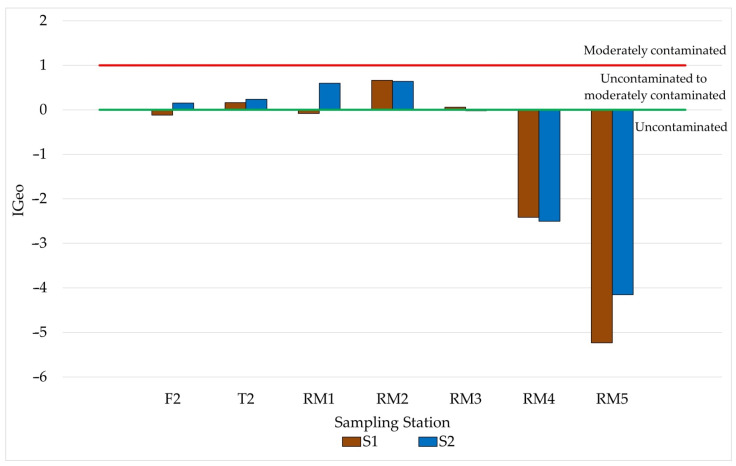
IGeo values in sediment samples collected in the Reis Magos River Hydrographic Basin.

In addition to the sediment quality assessment by the SQGs, the following pollution indices were calculated: IGeo, CF, and E_F_ [35,36,37,38,39,40]. Figure 5 shows the results found for IGeo, indicating that most samples showed moderate-to-non-contaminated contamination conditions. These results indicate little contamination due to anthropogenic activities in the studied region [34]. Station RM2, which has a more significant influence on agricultural activities (pasture, reforestation with eucalyptus, plantation of coffee, and sugar cane), showed higher values of IGeo [43]. The use of agrochemicals that contain Hg was banned in the 1980s; however, studies have shown the presence of products that are part of their composition, in 2019, in some regions of the country. Even after stopping the use of these agrochemicals, the region may have Hg contamination due to the persistence of this element in the environment, mainly associated with sediment particles [53].

**Figure 6:**
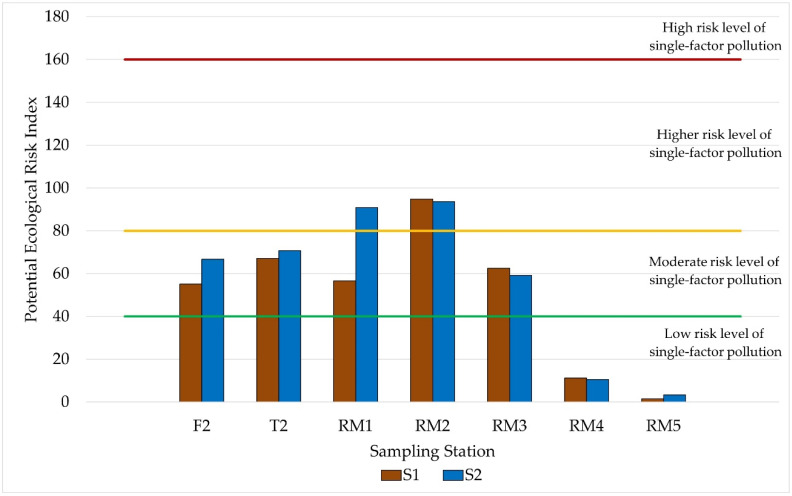
Values of the ecological risk potential index (E_F_) in the sediment samples collected in the Reis Magos River Hydrographic Basin.

When the contamination level was observed using the ecological risk potential index (E_F_), which takes into account the toxicity coefficient (T = 40 for Hg), it can be seen from Figure 6 that only RM4 and RM5 presented low levels of ecological risk.

RM1 (S1) and RM2 (S1 and S2) presented the highest values of ecological risk potential. This behavior was also observed for CF, with F2, T2, RM1, RM2, and RM3 showing a moderate contamination factor and RM4 and RM5 CF showing a low contamination factor (Table 3).

Observing the Hg concentrations found in this sample, it can be seen (Figure 2) that the values were below, but close to, the TEL value (170 µg∙kg^−1^). Evaluating the results obtained and the calculated IGeo and E_F_ pollution indices, it can be inferred that the region is moderately impacted concerning the presence of Hg in the sediment. This contamination can be associated with anthropic impacts, mainly from inadequate waste disposal and leaching of agricultural waste, such as fertilizers and pesticides.

Thus, the need for Hg monitoring is evidenced, especially in stations RM1 and RM2, which presented the highest Hg contents in the studied period. A more significant concern arose in 2017 when RMR, besides the water used for irrigation and fishing, became a source of urban supply. And after conventional water treatment, Hg is not considered in regular water quality monitoring schemes. 

## 4. Conclusions

With the results of the Hg concentration in water samples, it could be inferred that Hg has a low potential to cause adverse effects on biota and human health. However, sediment samples showed signs of moderate pollution (IGeo and E_F_), and because it is in direct contact with water, the transfer between these two matrices could be favored, considering the kinetics and the physical-chemical characteristics of the environment. Since Hg is a potential contaminant with a severe impact on the food chain, this study highlighted the need to monitor Hg in the RMRB, considering that the fluvial environment, particularly its lower course, has been used for irrigation and fishing activities, as well as being a source of water distribution to supply cities. Since there are few studies in this region, this paper could offer a base value for posterior works. Furthermore, this parameter can help assess the load of domestic and industrial effluents being dumped in this basin and reorder the occupations along its margin.

## Figures and Tables

**Table 1:** Geographical coordinates of sampling stations along the RMRB.

Sampling Stations	Designation	Geographic Coordinates
**Tributary rivers**		
F1	Fundão river spring	−19°49′23″/−40°26′24″
F2	Fundão river after housing	−19°59′28″/−40°19′44″
T1	Timbuí river spring	−19°57′4″/−40°40′41″
T2	Timbuí river after housing	−20°2′21″/−40°20′46″
**Reis Magos river**		
RM1	Confluence of the Timbuí and Fundão rivers (near the water distribution site for public supply)	−20°1′31″/−40°18′6″
RM2	Rural zone	−20°2′34″/−40°15′51″
RM3	Outflow of sewage treatment plant (SWTP)	−20°1′48″/−40°12′37″
RM4	Urban area	−20°2′49″/−40°11′25″
RM5	Reis Magos river mouth	−20°3′20″/−40°11′32″

**Table 2:** Lower and upper limits of Hg concentration for sediment and water according to national and international standards.

	Water		Water Sediment	
	Class 1 ^a^ and 2 ^a^	Class 3 ^a^	TEL^b^ (Level 1 ^c^)	PEL^b^ (Level 2 ^c^)
Reference	µg∙L^−1^		µg∙kg^−1^	
CCME, 2001 [29]; CONAMA, 2012 [32]	-	-	170	486
CONAMA, 2005 [28]	0.2	2	-	-
Buchman, 2008 [31]	0.77	1.4	174	486

Adapted from ^a^ [28], ^b^ [29,31] and ^c^ [32].

**Table 3:** Classification by value of geoaccumulation index (IGeo), contamination factor (CF), and potential ecological risk index (E_F_).

IGeo		CF		E_F_	
Range	Classification	Range	Classification	Range	Classification
>5	Extremely contaminated	≥6	Very high contamination factor	>320	serious
4–5	Strongly to extremely contaminated	3–6	Considerable contamination factor	160–320	high
3–4	Strongly contaminated	1–3	Moderate contamination factor	80–160	higher
2–3	Moderately to strongly contaminated	<1	Low contamination factor	40–80	moderate

Adapted from [33,34,35,36,37,38].

## Data Availability

https://quimica.vitoria.ufes.br/pt-br/pos-graduacao/PPGQUI/detalhes-de-pessoal?id=20719 (accessed on 26 September 2022).

## Supplementary Materials

The following supporting information can be downloaded at: https://www.mdpi.com/article/10.3390/ijerph191912626/s1, Figure S1: Water balance in Espírito Santo State in (a) S1 and (b) S2, Table S1: Physical-chemical characteristics and Hg concentration in the river water samples; Table S2: Physical-chemical characteristics and Hg concentration in the sediment samples.

## Abbreviations

AESB	Brazilian Association of State Sanitation Companies
AGERH	State Water Resource Agency
ANA	National Agency of Waters
CCME	Canadian Council of Ministers of the Environment
CF	Contamination Factor
CONAMA	Brazilian National Council for the Environment
CRM	Certified Reference Material
DO	Dissolved Oxygen
E_F_	Potential Ecological Risk Index
Embrapa	Brazilian Agricultural Research Corporation
ETE	Sewage Treatment Plant
F	Fundão River
IGeo	Geoaccumulation Index
Incaper	Capixaba Institute for Research, Technical Assistance and Rural Extension
LoD	Limits of Detection
LoQ	Limits of Quantification
NIST	National Institute of Standards and Technology
NOAA	National Oceanic and Atmospheric Administration
NSF	National Sanitation Foundation
OM	Organic Matter
ORP	Oxidation-Reduction Potential
PCA	Principal Component Analysis
PEL	Probable Effect Level
RM	Reis Magos River
RMRB	Reis Magos River Hydrographic Basin
RSD	Relative Standard Deviation
S1	Sampling 1
S2	Sampling 2
SQG	Quality Guidelines and Standards
T	Timbuí River
TDA AAS	Thermodecomposition and Amalgamation Atomic Absorption Spectrometry
DMA	Direct Mercury Analyzer
TDS	Total Dissolved Solids
TEL	Threshold Effect Concentration
WQI	Water Quality Indices
