# Supplementary material for: Hg Pollution Indices along the Reis Magos River Basin—Brazil: A Precursory Study
Source: Int J Environ Res Public Health. 2022 Oct 3;19(19):12626. doi: 10.3390/ijerph191912626 (PMC9564948; doi:10.3390/ijerph191912626)
Supplement: Supplementary file 1 [file ijerph-19-12626-s001.zip › ijerph-1896283-supplementary.pdf]

Supplemental Material

Table S1. Physical-chemistry characteristics and Hg concentration in the river water samples.

Sampling station (sampling)	Hg concentration	T	pH	Electric conductivity	ORP	Turbidity	DO	STD	Salinity
	μg·L⁻¹	ºC	-	mS·cm⁻¹	mV	NTU	mg·L⁻¹	g·L⁻¹	ppt
F1T (S1)	<0.14	26.29	8.01	0.044	298	2.6	12.38	0.029	0.0
T1T (S1)	<0.14	20.75	7.19	0.059	317	0	11.7	0.115	0.0
F2T (S1)	<0.14	25.80	7.21	0.177	344	2.3	13.85	0.039	0.1
T2T (S1)	<0.14	25.83	7.34	0.106	262	3.4	14.67	0.07	0.0
RM1T (S1)	<0.14	25.55	7.67	0.128	279	5.3	11.98	0.044	0.0
RM2T (S1)	<0.14	26.42	7.45	0.23	302	7.2	10.65	0.054	0.0
RM3T (S1)	<0.14	26.51	7.88	7.21	311	18.6	8.74	0.205	0.1
RM4T (S1)	<0.14	26.68	7.80	19.98	305	20.1	10.02	0.55	0.2
RM5T (S1)	<0.14	25.81	7.92	24.5	299	19.8	9.54	1.91	0.3
F1T (S2)	<0.14	27.24	7.65	0.047	273	1.9	10.22	0.031	0.0
T1T (S2)	<0.14	28.12	7.55	0.071	291	17.1	11.93	0.034	0.0
F2T (S2)	<0.14	21.23	5.50	0.053	379	0	10.79	0.046	0.0
T2T (S2)	<0.14	27.69	6.60	0.081	240	10.1	13.02	0.053	0.0
RM1T (S2)	<0.14	27.98	7.95	0.072	261	27.5	7.08	0.047	0.0
RM2T (S2)	<0.14	28.19	5.99	0.095	315	22.2	5.30	0.062	0.0
RM3T (S2)	<0.14	28.76	6.24	0.223	313	29.3	4.07	0.145	0.1
RM4T (S2)	<0.14	29.03	6.83	0.327	268	36.3	8.69	0.212	0.2
RM5T (S2)	<0.14	29.07	7.28	0.404	252	39.9	6.99	0.264	0.2

Table S2: Physical-chemistry characteristics and Hg concentration in the sediment samples.

					Granulometric content			
**Sampling station (sampling)**	**Hg concentration ± Standard deviation**	**pH**	**OM**	**Carbonate**	**Gravel**	**Sand**	**Silt**	**Clay**
	µg·kg^{-1}	-	%	%	%	%	%	%
F2 (S1)	73.54 ± 0.41	3.81	2.91	1.34	22.38	67.22	8.89	1.51
T2 (S1)	91.04 ± 1.33	6.72	1.30	2.02	4.71	85.19	7.51	2.59
RM1 (S1)	75.71 ± 1.62	5.14	2.30	0.34	9.72	67.8	18.08	4.40
RM2 (S1)	135.18 ± 0.47	5.91	22.31	0	1.57	5.06	70.02	23.35
RM3 (S1)	84.18 ± 0.10	6.56	22.44	2.23	3.75	17.23	63.22	15.80
RM4 (S1)	16.04 ± 0.35	7.8	10.99	39.39	2.18	6.99	89.61	1.22
RM5 (S1)	4.97 ± 0.11	6.97	2.82	13.89	39.81	41.46	16.05	2.68
F2 (S2)	93.47 ± 3.07	4.55	3.22	1.48	19.38	75.04	5.09	0.49
T2 (S2)	98.95 ± 2.21	5.92	2.34	2.26	8.71	87.19	3.75	0.35
RM1 (S2)	127.16 ± 2.41	4.78	5.90	0.43	13.5	54.62	25.9	5.98
RM2 (S2)	131.08 ± 1.06	4.56	20.99	0	3.51	10.56	71.82	14.11
RM3 (S2)	82.86 ± 1.02	7.02	17.63	1.11	19.64	26.38	46.26	7.72
RM4 (S2)	14.78 ± 1.32	7.22	11.81	20.98	1.22	28.57	58.51	11.70
RM5 (S2)	4.71 ± 0.18	6.46	3.78	11.85	23.49	63.33	12.89	0.29

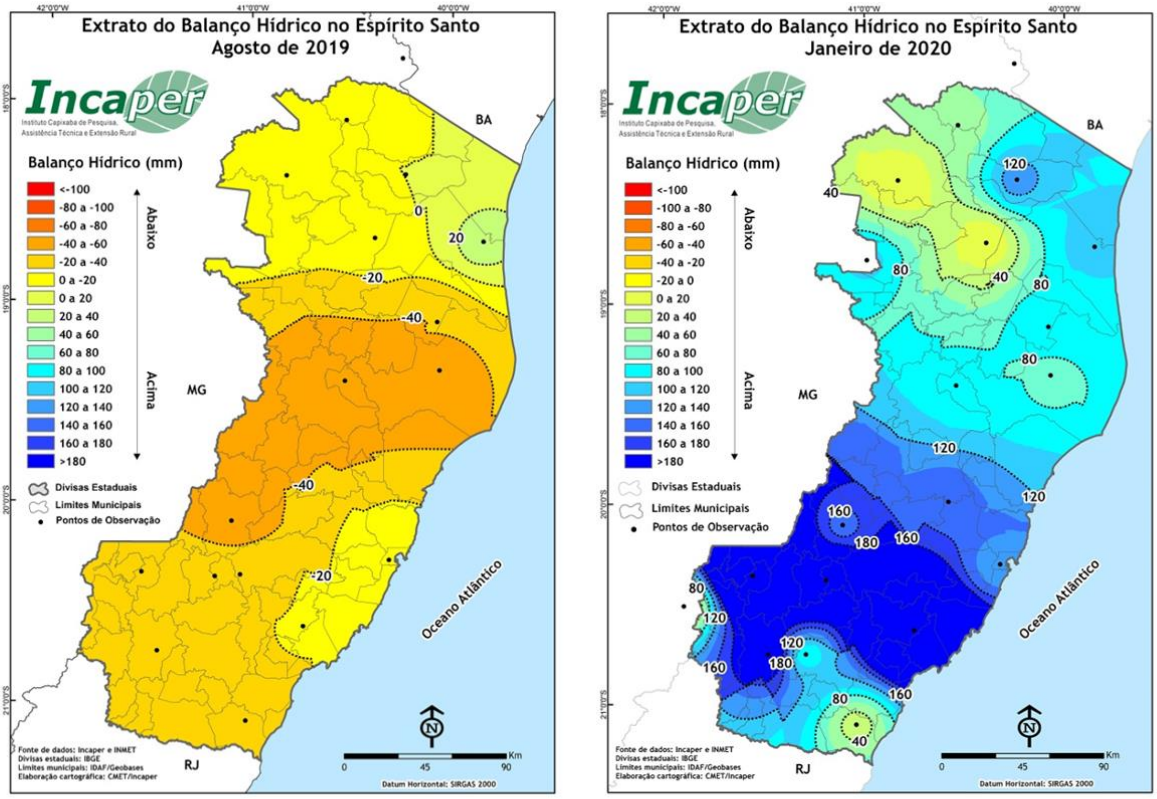

(**a**) (**b**)

Figure S1. Water balance in Espírito Santo State in (**a**) S1 and (**b**) S2.
